# Effects of Personality Styles on Clinical Response to Intermittent Theta Burst Stimulation for Depression

**DOI:** 10.3390/jcm14217612

**Published:** 2025-10-27

**Authors:** Mohamed A. Abdelnaim, Tobias Hebel, Katharina Kerkel, Berthold Langguth, Martin Schecklmann, Susanne Staudinger, Andreas Reissmann

**Affiliations:** Department of Psychiatry and Psychotherapy, University Regensburg, 93053 Regensburg, Germany; mohamed.abdelnaim@medbo.de (M.A.A.); katharina.kerkel@medbo.de (K.K.); berthold.langguth@medbo.de (B.L.); martinschecklmann@gmail.com (M.S.); andreas.reissmann@medbo.de (A.R.)

**Keywords:** TMS, iTBS, depression, personality disorders, TMS outcome

## Abstract

**Introduction:** Major depressive disorder (MDD) is a common and often treatment-resistant condition, with many patients showing only partial or minimal response to standard therapies. Repetitive transcranial magnetic stimulation (rTMS) is a well-established, non-invasive treatment for depression, though individual response varies considerably. While demographic and clinical predictors have been explored, the impact of personality styles on rTMS outcomes remains underinvestigated. Herein, we aimed to explore whether personality styles influence treatment response to rTMS. **Methods:** This retrospective study included 63 in- and outpatients with depressive episodes treated with intermittent theta-burst stimulation (iTBS) between September 2020 and December 2022. Patients were assessed before and after treatment using the 21-item Hamilton Depression Rating Scale (HAMD-21) and the self-reported Major Depression Inventory (MDI). Personality styles were evaluated using the German *Persönlichkeits-Stil-und-Störungs-Inventar* (PSSI), a dimensional measure of 14 personality styles. Statistical analyses included paired-samples *t*-tests to assess symptom change and linear regression models to examine whether personality styles predicted treatment outcomes. Effect sizes were reported as Cohen’s *d*. **Results:** Patients showed a significant reduction in depressive symptoms following iTBS (HAMD-21: *t*(62) = 10.86, *p* < 0.001, *d* = 1.37. MDI: *t*(62) = 8.55, *p* < 0.001, *d* = 1.06). Stepwise regression for the MDI identified critical–negativistic (NT) and reserved–schizoid (SZ) styles as significant predictors, explaining approximately 16% of the variance (*R*^2^ = 0.159, *p* = 0.007). When entered simultaneously in a regression model for the HAMD-21, these same traits also predicted symptom change, though the effect was smaller (*R*^2^ = 0.108, *p* = 0.033). Higher scores of critical–negativistic (NT) style were associated with better improvement, whereas higher scores of reserved–schizoid (SZ) style were associated with less improvement. **Conclusions:** This study confirms the overall efficacy of rTMS in reducing depressive symptoms. While SZ and NT traits showed some predictive value for treatment response—particularly on self-reported outcomes—their influence was modest and inconsistent. Based on our findings, there is no reason why patients with depression and specific personality styles, or even comorbid personality disorders, should be denied rTMS treatment.

## 1. Introduction

Depression is a common mental health disorder [1,2], with a reported lifetime prevalence of 10.8% [3]. It is often chronic and significantly diminishes an individual’s professional capabilities [4] and overall quality of life [5]. In its most extreme manifestation, depression can result in suicide [6] and increased mortality risk [7]. Depression ranks as a primary cause of disability and significantly contributes to the global burden of disease [8].

Despite the efficacy of drugs, psychotherapy, and electroconvulsive therapy (ECT) in treating depression, a considerable proportion of patients do not achieve a complete response to the available interventions [9]. Nearly fifty percent of patients with depression persist in exhibiting residual depressive symptoms after receiving appropriate treatment, and up to twenty percent may have minimal or no response to even the most intensive therapies [10,11]. Furthermore, some patients cannot tolerate the presently available therapies, and among those who can tolerate them, the duration to response may extend over several weeks, and relapse is frequent [12]. This has resulted in a growing search for alternatives, including various brain stimulation techniques, like vagus nerve stimulation, transcranial magnetic stimulation, and deep brain stimulation [13].

Repetitive transcranial magnetic stimulation (rTMS) is a non-invasive brain stimulation method that has developed over the past 40 years and is utilized for the treatment of numerous neuropsychiatric disorders. Repetitive transcranial magnetic stimulation (rTMS) employs pulsed magnetic fields with an intensity of up to 3 Tesla to provoke neuronal depolarization in superficial cortical regions [14].

In 2008, the FDA approved rTMS as a treatment for severe depression in individuals refractory to at least one antidepressant during the current episode [15] (FDA approval K061053). In Europe, rTMS has already received CE certification and is approved for the treatment of major depressive episodes [16], with national guidelines (e.g., Germany and the United Kingdom) recommending it as a clinically effective and safe non-invasive therapeutic option [17,18].

The effectiveness of rTMS has been demonstrated by numerous double-blind, randomized controlled studies [15,19,20] and has been reported in various systematic meta-analyses, reviews [21,22,23], and evidence-based guidelines [24]. However, rTMS effects vary highly among patients, with some achieving significant benefits or remission and others exhibiting minimal or no response. The reasons for the varying efficacy are still incompletely understood.

Previous studies have identified several factors influencing response to rTMS in depression, including clinical characteristics such as illness duration, treatment refractoriness, and symptom profiles [25,26,27]. Psychological and behavioral factors—such as motivation, cognitive flexibility, and comorbid personality pathology—have also been proposed as potential moderators of treatment outcome [28,29,30]. However, the specific role of personality dimensions in shaping rTMS response remains largely unexplored, despite evidence that personality pathology can affect outcomes in pharmacological and psychotherapeutic interventions [31,32,33].

For depression, the most prevalent comorbidities are anxiety disorders, substance use disorders, other depressive disorders, and personality disorders [34]. Patients with comorbid personality disorders have an elevated risk of persistence of depression [35], a poorer response to treatment [36,37], and a higher risk of suicide attempts [38]. The influence of personality disorders on the effectiveness of Transcranial Magnetic Stimulation (TMS) for depression remains an underexplored area in current research.

In this study, we approached the relevance of co-morbid personality disorders for the antidepressant efficacy of rTMS. With respect to the assessment of co-morbid personality disorders, we considered a dimensional approach more appropriate than a categorical approach and therefore used an instrument for assessing personality styles. Personality styles describe variations in an individual’s personality instead of categorizing them as normal and pathological. Thus, we investigated whether the individual expression of various personality styles has an impact on the antidepressant efficacy of TMS treatment.

## 2. Materials and Methods

### 2.1. Study Design and rTMS-Protocol

This study was approved by the local ethics committee of the University of Regensburg (20-1932-101). All patients provided written informed consent for the treatment as well as for data collection and analysis. The study was carried out in the context of clinical routine treatment. All patients underwent rTMS treatment at the Bezirksklinikum Regensburg (Germany) between September 2020 and December 2022, with treatment courses lasting either three or six weeks. All patients referred to the TMS unit were interviewed by a psychiatrist or clinical psychologist with experience in brain stimulation to evaluate the indication, possible contraindications of rTMS treatment, and also the eligibility of the patient to participate in the study.

All patients were treated with intermittent theta-burst stimulation (iTBS). The rTMS sessions were administered and monitored by trained psychiatric nursing staff or physician assistants. Stimulation was applied to the left dorsolateral prefrontal cortex (DLPFC), localized according to the F3 position of the international 10–20 EEG system [39]. Treatments were delivered using a MagVenture system (MagVenture A/S, Farum, Denmark) equipped with a figure-of-eight coil and the default current direction. The stimulation intensity was targeted at 120% of the resting motor threshold (RMT). The RMT was determined by applying single magnetic pulses over the left motor cortex and identifying the lowest output intensity that evoked a measurable motor-evoked potential (MEP) in the corresponding finger muscle, recorded via electromyography (EMG) [40]. In patients with an RMT ≥ 50% of the maximal stimulator output (MSO), a stimulation intensity of 60% MSO was chosen. In cases where patients experienced local discomfort, the stimulation strength was individually adjusted to the highest tolerable intensity to maintain comfort throughout the session.

### 2.2. Patients and Measures

The study described herein is a retrospective analysis of in- and outpatients who were treated with rTMS because of a depressive episode. All patients registered for a TMS treatment at the Bezirksklinikum Regensburg during the study period were invited to participate in our study (TMS Plus), which comprised several assessment modules, including a module involving completion of the German questionnaire *Persönlichkeits-Stil-und-Störungs-Inventar* (engl. Personality Style and Disorder Inventory (PSSI); [41]. Of a total of 595 patients who underwent TMS treatment between September 2020 and December 2022, 92 patients (15.5%) provided written informed consent to participate in the TMS-Plus study. Among these, 63 patients met eligibility criteria and were included in the present analysis. Participation was entirely voluntary and had no impact on the patients’ clinical treatment or therapeutic procedures.

The PSSI questionnaire consists of 140 items grouped into 14 subscales, with each scale representing a distinct personality style. These styles are conceptually related to the personality disorders listed in major diagnostic manuals but are assessed dimensionally rather than categorically: self-willed–paranoid (PN), reserved–schizoid (SZ), suspicious–schizotypal (ST), spontaneous–borderline (BL), amiable–histrionic (HI), ambitious–narcissistic (NA), assertive–antisocial (AD), self-critical–self-insecure (SU), loyal–dependent (AB) meticulous–compulsive (ZW), critical–negativistic (NT), silent–depressive (DP), helpful–selfless (SL), and optimistic–rhapsodic (RH) (the names of the subscales were translated literally). Patients rated each item on a 4-point Likert scale, assessing the degree to which a statement applies to them. The scale is as follows: 0—not at all true, 1—mostly not true, 2—mostly true, and 3—completely true. Item scores were added to form 14 different subscale scores, which were translated to T scores based on normative data [41]. The subscales have acceptable to good levels of internal consistency (α ranges between 0.73 and 0.85).

**Figure 1:**
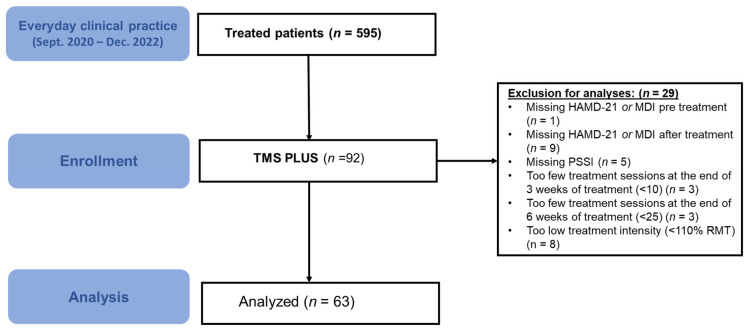
**CONSORT flow diagram.** Flow-chart showing the total number of patients and exclusion criteria for the analysis of the TMS Plus study.

In the present study, we focused on the question of whether personality styles, according to the PSSI score, had an impact on the antidepressant effect of TMS treatment. Depressive symptoms were assessed with the Hamilton Depression Rating Scale (HAMD-21) [42] and Major Depression Inventory [43], before the beginning and after the end of rTMS treatment. The number of enrolled and analyzed patients, as well as reasons for exclusion, can be found in the CONSORT Flow Diagram (Figure 1). 

Because this was a retrospective clinical dataset, detailed information on psychotropic medication (type, stability, or changes), concurrent psychotherapy, and clinical characteristics such as treatment resistance level, and comorbid anxiety was not consistently available. Consequently, these potential confounders could not be systematically controlled for in the statistical analyses.

### 2.3. Statistical Analysis

All statistical analyses were performed using SPSS version 29.0 (IBM SPSS, Chicago, IL, USA). Since two depression scales were analyzed, the significance threshold was adjusted for multiple comparisons using Bonferroni’s correction (*p* = 0.025). For demographic and clinical data, mean values (M) and standard deviations (SD) are reported. A paired samples *t*-test was calculated for the course of depressive symptoms. Therefore, the following values were used: baseline values and the respective final values regardless of the total duration of treatment (3 or 6 weeks). To examine the predictive value of personality dimensions on changes in depressive symptoms, linear regression analyses were conducted using change scores (final—baseline score) for both the HAMD-21 and MDI as dependent variables and the 14 subscales of the PSSI as potential predictors. First, two-stepwise linear regression models were conducted to identify the most parsimonious set of predictors for all 14 PSSI subscales. Variable entry and removal were based on the probability of F, with an entry threshold of *p* < 0.05 and a removal threshold of *p* > 0.10. For the HAMD-21, we subsequently specified a regression model using the enter method, employing the set of predictors identified in the MDI model as fixed predictors (i.e., subscale scores of the *reserved–schizoid* and *critical–negativistic* subscales). Model assumptions regarding linearity, multicollinearity among predictors, homoscedasticity, and normality of residuals were examined. The normal P–P plot indicated no substantial deviations, and the Shapiro–Wilk test confirmed that the standardized residuals were normally distributed for both the HAMD-21 (W = 0.98, *p* = 0.20) and the MDI model (W = 0.98, *p* = 0.41).

For effect sizes, we used Cohen’s *d* [44]. By convention, effect sizes are divided in small (*d* = 0.2; partial η^2^ = 0.01; φ = 0.1), medium (*d* = 0.5; partial η^2^ = 0.06; φ = 0.3), and large effects (*d* = 0.8; partial η^2^ = 0.14; φ = 0.5). The demographic and clinical data of the included patients are provided in Table 1.

## 3. Results

For the clinical scale scores, paired samples *t*-tests both regarding the HAMD-21 data (*t*(62) = 10.86, *p* < 0.001, *d* = 1.37) as well as the MDI data (*t*(62) = 8.55, *p* < 0.001, *d* = 1.06) revealed statistically significant and large reductions in depressive symptoms following the TMS treatment.

**Figure 2:**
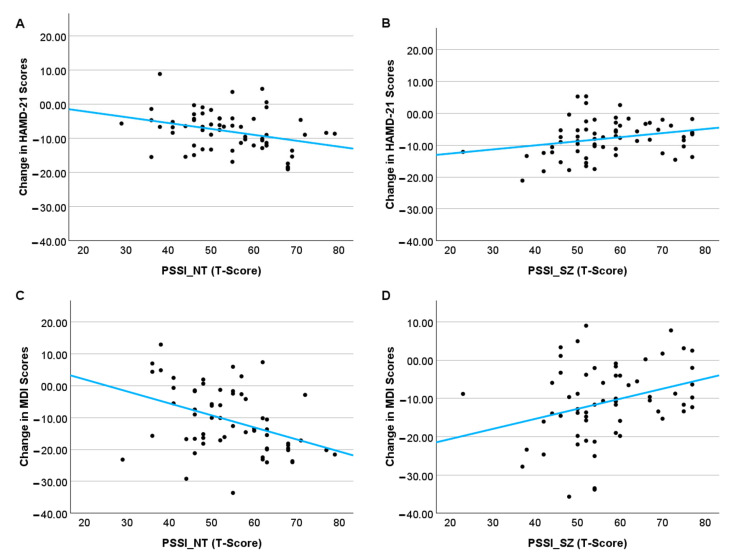
**Association between PSSI subscale scores and clinical outcome.** (**A**) Change in HAMD-21 scores by “critical–negativistic” (PSSI-NT) trait; (**B**) change in HAMD-21 scores by “reserved–schizoid” (PSSI-SZ) trait; (**C**) change in MDI scores by “critical–negativistic” (PSSI-NT) trait; (**D**) change in MDI scores by “reserved–schizoid” (PSSI-SZ) trait; regression lines illustrate model-based associations adjusted for the respective other subscale; note that negative change scores point towards symptom reduction during the course of treatment.

When using the stepwise method to predict change scores in depressive symptoms, no significant predictors could be identified for the HAMD-21 and therefore, no model could be retained during the step of the analytic procedure. Regarding the MDI, two predictors, “reserved–schizoid SZ” (*β* = 0.299, *p* = 0.026) and “critical–negativistic NT” (*β* = −0.405, *p* = 0.003) were retained in the final model as significant predictors of treatment outcome. This model was statistically significant (F(2, 62) = 5.471, *p* = 0.007), accounting for 15.9% of the variance in MDI change scores (*R* = 0.398, *R*^2^ = 0.159, adjusted *R*^2^ = 0.130). When rerunning a regression model with these two predictors for the HAMD-21 change scores, a significant model could be identified (F(2, 62) = 3.616, *p* = 0.033), explaining 10.8% of the variance in HAMD-21 change scores (*R* = 0.328, *R*^2^ = −108, adjusted *R*^2^ = 0.078). The PSSI subscale “critical–negativistic NT” (*β* = −0.327, *p* = 0.016) significantly impacted HAMD-21 change scores, while the influence of the “reserved–schizoid SZ” subscales (*β* = 0.256, *p* = 0.058) was only marginally significant. Figure 2 shows the predicted course of depression depending on PSSI subscale trajectories for both the HAMD-21 (Figure 2A,B) and the MDI (Figure 2C,D).

Table 2 provides correlations between PSSI subscales and change scores on the HAMD-21 and MDI.

## 4. Discussion

Depression is a prevalent psychiatric illness that often occurs in combination with other psychiatric disorders [45]. Previous research has demonstrated that comorbid personality pathology can influence the effectiveness of depression treatments [31,32].

The present study confirms that rTMS is an effective intervention for reducing depressive symptoms, measured by the HAMD-21 and MDI, supporting rTMS as a robust intervention for major depressive episodes and consistent with prior meta-analyses [24,46].

Beyond confirming general effectiveness, this study aimed to investigate whether individual personality styles or disorders, assessed dimensionally via the Personality Style and Disorder Inventory (PSSI), might influence the antidepressant response to rTMS. This is clinically relevant, as personality pathology has been linked to slower or less complete responses to treatments for depression. Previous research has suggested a potential influence of personality disorders on treatment outcomes in depression, including response to pharmacotherapy, psychotherapy, and neuromodulation techniques such as rTMS [33,47].

Regression analyses using baseline PSSI scores did not reveal significant predictors of symptom change on the HAMD-21 when applying the stepwise method. However, when NT and SZ were entered simultaneously (based on the MDI model), a significant model emerged, with higher scores on the “critical–negativistic” (NT) scale associated with greater symptom improvement, and higher scores on the “reserved–schizoid” (SZ) scale associated with marginally less improvement. For the MDI, stepwise regression identified both reserved–schizoid (SZ) and critical–negativistic (NT) traits as significant predictors, together accounting for approximately 16% of the variance in symptom improvement. Although these findings suggest a potential influence of personality styles/disorders on treatment response, the size of bivariate correlation coefficients across PSSI subscales was in the negligible-to-small effect range in all cases (<0.30, see Table 2).

These findings diverge from earlier studies, which suggested that personality dimensions may profoundly shape treatment trajectories, although findings are often mixed and inconsistent [48,49,50]. Traits associated with psychological well-being—such as autonomy, self-directedness, and personal growth—have been linked to more favorable treatment outcomes [51]. For example, higher scores on the self-directedness scale have been associated with greater clinical improvement in treatment-resistant depression, while other traits have shown less consistent predictive value [52]. Similarly, research using the NEO-PI-R model has indicated that extraversion [53], agreeableness and conscientiousness [54], and persistence [55] may serve as positive predictors of response to rTMS. These personality dimensions are thought to reflect behavioral flexibility, motivation, and social engagement—factors that may interact with neural circuits targeted by rTMS [56]. Neuroimaging research has further suggested that personality-related traits may influence brain connectivity in ways relevant to rTMS mechanisms. For instance, harm avoidance has been associated with reduced connectivity between the default mode network and the subgenual anterior cingulate cortex (sgACC)—a region repeatedly implicated in rTMS treatment response [57]. Additionally, personality vulnerabilities such as chronic pessimism and learned helplessness have been linked to treatment resistance in depression, possibly through their impact on affect regulation and cognitive reactivity [58].

While these studies support a theoretical role for personality traits in moderating rTMS effects, our current findings provide only limited empirical support for profound associations between personality and TMS effects. These results underscore the complexity of identifying reliable psychological predictors of rTMS response.

The association of reserved–schizoid (SZ) and critical–negativistic (NT) traits with changes in depressive symptoms may reflect specific psychological mechanisms that influence both subjective and observable aspects of treatment response. Individuals with a schizoid personality style often exhibit emotional detachment, social withdrawal, and limited capacity for introspection [59]. These traits may reduce emotional engagement with treatment or result in a diminished expectation of improvement, potentially affecting how therapeutic gains are perceived and reported. In contrast, those with a critical–negativistic style may display chronic skepticism, mistrust, and passive-aggressive tendencies. Such individuals might be more prone to critical appraisals of therapeutic interventions, potentially undervaluing actual benefits [60]. However, our findings indicate that individuals with higher negativistic traits nonetheless reported meaningful symptom relief following rTMS. One possible explanation is that their low expectations of therapeutic benefit may have heightened the perceived impact of actual improvement when it occurred. Additionally, the external and ‘non-intrusive’ nature of rTMS might circumvent psychological resistance typically associated with more interpersonal treatment settings. Biologically, individuals with chronic critical affect may also exhibit distinct patterns of frontolimbic dysregulation—patterns that rTMS is particularly well-suited to modulate.

These observations suggest that personality traits may modulate aspects of treatment response and highlight the value of further exploratory research in personality-stratified rTMS settings.

Nonetheless, the small and partially inconsistent associations across employed depression scales suggest that personality styles, while theoretically relevant, may not play a robust or straightforward role in predicting rTMS treatment response, at least not in isolation.

Our findings highlight the need for caution in interpreting the role of personality in predicting rTMS response. Future research should employ harmonized outcome measures, larger sample sizes, and possibly multi-modal approaches (e.g., combining personality assessment with neuroimaging or physiological markers) to clarify whether specific personality dimensions interact meaningfully with neuromodulation-based interventions. Moreover, research should consider longer-term outcomes and the potential interaction of personality traits with relapse risk or maintenance treatment strategies. Nevertheless, our findings are highly relevant from a clinical perspective, as they clearly demonstrate that patients with depression and specific personality traits, or even comorbid personality disorders, should not be denied rTMS treatment.

In conclusion, while rTMS demonstrates strong efficacy in alleviating depressive symptoms, personality styles appear to have limited and inconsistent predictive value in this context. Nevertheless, further exploration of psychological moderators remains warranted.

## 5. Limitations

A key limitation of this study is the relatively small sample size (N = 63), which limits statistical power and increases the risk of model instability and overfitting, especially given the number of predictor variables tested. Consequently, our regression results should be regarded as preliminary and interpreted with caution. The observed associations require confirmation in larger, prospectively designed samples with greater statistical power. Additionally, due to incomplete clinical records, we were unable to systematically control for confounding variables such as medication use, concurrent psychotherapy, degree of treatment resistance, or comorbid psychiatric conditions. These factors are well known to influence treatment outcomes in rTMS and may have contributed to variability in our findings.

Furthermore, the study included patients with various personality styles, with limited variability for the various personality dimensions. The interaction between the various personality styles may have influenced the observed effects, making it difficult to draw definitive conclusions about the impact of individual personality styles or disorders on TMS outcomes and, overall, this may limit the generalizability of our findings to broader clinical populations. Additionally, personality assessment was based on a single assessment tool, which, while validated, relies on self-report and is less comprehensive than instruments providing a more detailed assessment of personality. Also, the study primarily assessed treatment outcomes within a limited follow-up period.

## Figures and Tables

**Table 1:** Demographic and clinical data of the present sample.

		Value (*n* = 63)
**General variables**		
Sex: m/f		27/36
Age: M (SD)		39.24 (12.67)
Age: range		18–63
**TMS variables**		
No. of sessions: M (SD)		20.91 (1.20)
Resting motor threshold (%): M (SD)		39.98 (6.93)
Intensity of treatment (%): M (SD)		47.84 (7.74)
**Depression questionnaire scores: M (SD)**		
**HAMD-21**	Baseline	19.95 (5.14)
	After treatment	12.04 (5.80)
**MDI**	Baseline	32.27 (8.24)
	After treatment	21.16 (10.55)
**PSSI subscales**		
Self-willed–paranoid (PN)		52.11 (12.86)
Reserved–schizoid (SZ)		57.10 (11.55)
Suspicious–schizotypal (ST)		44.29 (6.70)
Spontaneous–borderline (BL)		58.98 (8.00)
Amiable–histrionic (HI)		44.38 (9.80)
Ambitious–narcissistic (NA)		45.19 (8.74)
Self-critical–self-insecure (SU)		59.81 (8.49)
Loyal–dependent (AB)		54.44 (9.85)
Meticulous–compulsive (ZW)		54.70 (10.29)
Critical–negativistic (NT)		54.11 (10.90)
Silent–depressive (DP)		64.49 (9.27)
Helpful–selfless (SL)		55.67 (9.43)
Optimistic–rhapsodic (RH)		40.56 (8.69)
Assertive–antisocial (AS)		41.62 (9.19)

**Table 2:** Pearson Correlations (r) between all PSSI subscales and change questionnaire scores (HAMD-21 and MDI).

Course of the Symptoms	PN	SZ	ST	BL	HI	NA	SU	AB	ZW	NT	DP	SL	RH	AS
**HAMD-21**	0.028	0.129	0.102	−0.016	−0.050	−0.065	0.191	0.102	0.063	−0.228	0.047	0.067	0.010	−0.094
**MDI**	−0.068	0.141	0.173	−0.066	−0.151	−0.155	0.054	−0.030	<0.001	−0.288 *	−0.239	0.044	−0.096	−0.106

*Notes.* * indicates a significant Pearson Correlation (*p* < 0.025). Self–willed-paranoid (PN), reserved–schizoid (SZ), suspicious–schizotypal (ST), spontaneous–borderline (BL), amiable–histrionic (HI), ambitious–narcissistic (NA), self-critical–self-insecure (SU), loyal–dependent (AB), meticulous–compulsive (ZW), critical–negativistic (NT), silent–depressive (DP), helpful–selfless (SL), optimistic–rhapsodic (RH), and assertive–antisocial (AS).

## Data Availability

The data used in this article can be requested from the corresponding author.
